# Single-incision laparoscopic surgery for intestinal intussusception due to neuroendocrine tumor

**DOI:** 10.1186/s40792-023-01639-2

**Published:** 2023-04-09

**Authors:** Toshinori Sueda, Mitsuyoshi Tei, Soichiro Mori, Kentaro Nishida, Akinobu Yasuyama, Yukihiro Yoshikawa, Masatoshi Nomura, Chikato Koga, Hiromichi Miyagaki, Masanori Tsujie, Yusuke Akamaru

**Affiliations:** Department of Gastroenterological Surgery, Osaka Rosai Hospital, 1179-3 Nagasone-Kitaku, Sakai City, Osaka 591-8025 Japan

**Keywords:** Intestinal intussusception, Neuroendocrine tumor (NET), Single-incision laparoscopic surgery (SILS)

## Abstract

**Background:**

Small intestinal neuroendocrine tumor (NET) is uncommon, but intestinal intussusception caused by NET is even rare. We report a rare case of single-incision laparoscopic surgery (SILS) for intestinal intussusception due to NET G1.

**Case presentation:**

A 72-year-old woman presented with vomiting, diarrhea, and abdominal pain. Contrast-enhanced computed tomography (CT) revealed the target sign in the ascending colon. An enhanced nodule was detected at the lead point, leading us to suspect a tumor. Colonoscopy showed a tumor at the lead point of the intestinal intussusception. Histological findings led to a diagnosis of NET G1. Single-incision laparoscopic ileocecal resection with regional lymphadenectomy was then performed. The patient was discharged 10 days postoperatively with no complications.

**Conclusion:**

We achieved SILS with regional lymphadenectomy for preoperatively diagnosed intestinal intussusception due to NET G1. Although this condition is rare, surgeons should take this possibility into consideration in cases showing similar findings.

## Background

Adult intestinal intussusception represents 5% of all intestinal intussusceptions and 1–5% of adult bowel obstructions [1]. Adult intestinal intussusception is normally preceded by a lead point [1]. When intestinal intussusception occurs, the mesentery is dragged into the bowel. This results in congestion of the lymphatic and vascular drainage systems and subsequent bowel edema, leading to bowel ischemia, perforation, and peritonitis. Surgical resection is therefore recommended due to the risk of emergency or malignancy. On the other hand, neuroendocrine tumors (NETs) of the small intestine represent a subgroup of gastroenteropancreatic NETs (GEP-NETs) [2]. All GEP-NETs were categorized into well-differentiated NETs, poorly differentiated neuroendocrine carcinomas, and mixed endocrine/non-endocrine neoplasms [3]. Well-differentiated NETs were further divided into grades G1, G2, and G3 [3]. Single-incision laparoscopic surgery (SILS) that further minimizes the surgical trauma is a challenging technique, because many difficulties come with operating within a confined operating space. However, the benefits of SILS are good cosmesis, less postsurgical pain, acceleration of recovery, or shorter length of postsurgical stay in comparison to multiport laparoscopic surgery (MIS) [4–7]. We report our experience with adult intestinal intussusception due to NET G1 resected using SILS.

## Case presentation

**Fig. 1:**
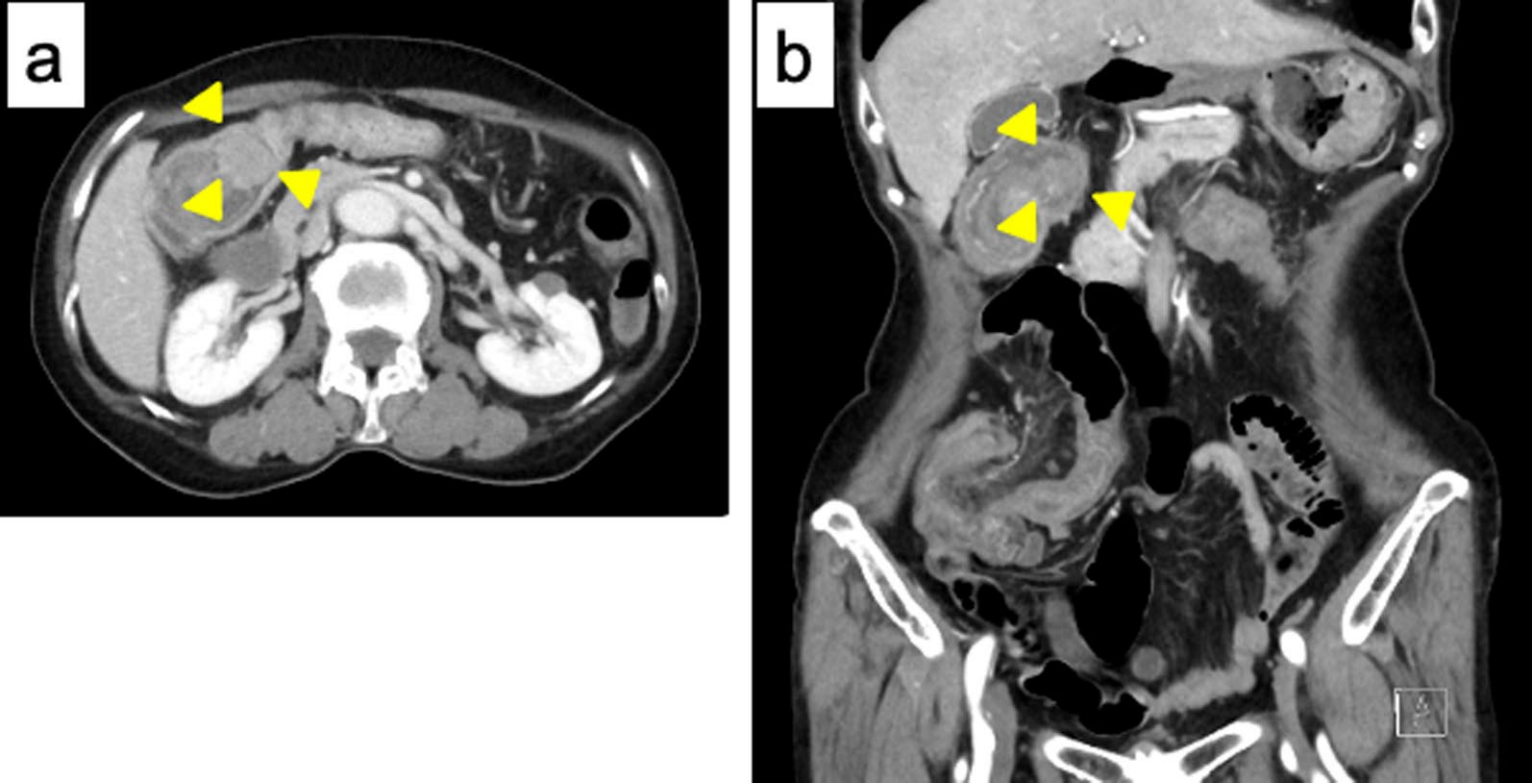
An abdominal contrast CT scan. Target sign is detected in the ascending colon in right upper abdomen, which indicates intussusception.** a** Axial views. **b** coronal views. Arrowhead: a lead point

**Fig. 2:**
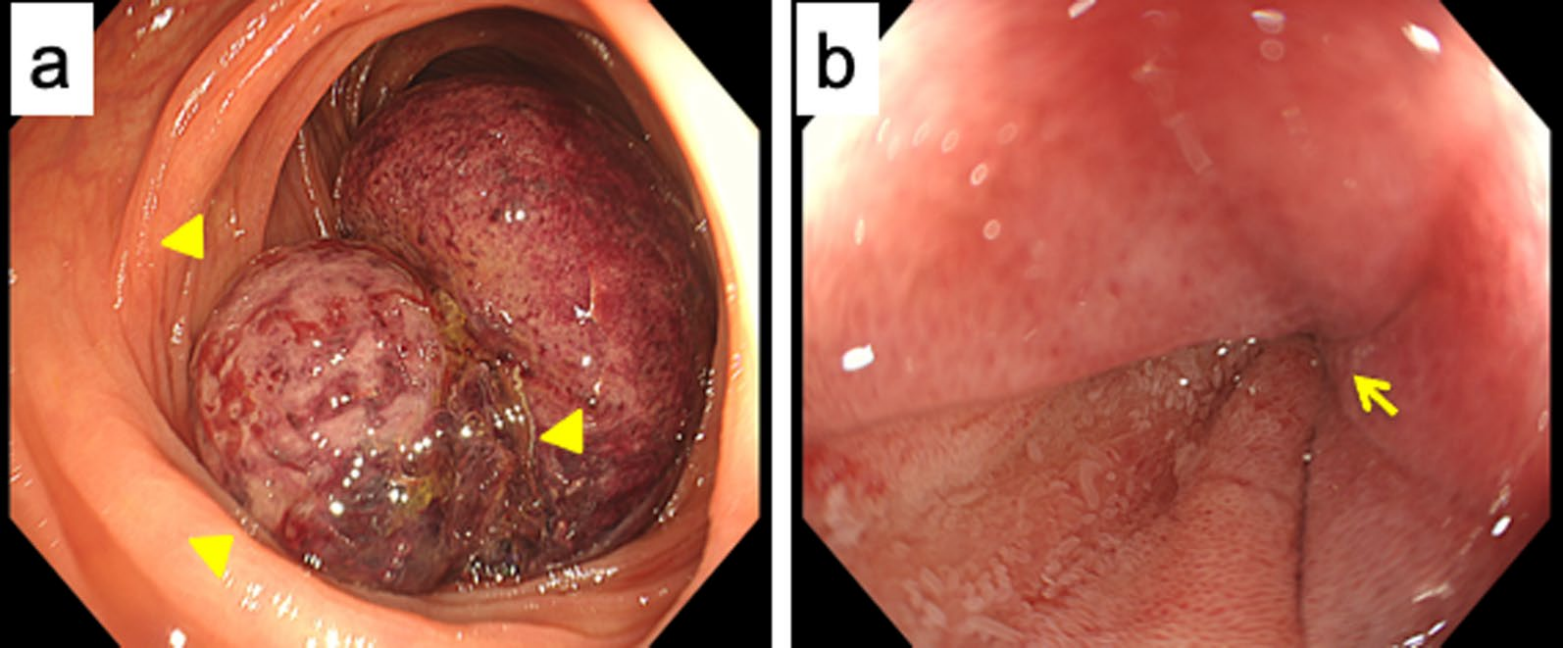
**a** Preoperative colonoscopy shows the existence of a tumor at the lead point of the intussusception (arrowhead). **b** There was evidence of bowel obstruction (arrow) and bowel wall edema

A 72-year old woman presented with a 10-day history of vomiting, diarrhea, and abdominal pain. She had no relevant medical history and no family history of malignancy. Physical examination showed a mass over the right abdominal region, but palpation elicited no significant abdominal tenderness. Laboratory tests were within normal ranges, except for a slightly increased level of C-reactive protein (0.70 mg/dL). Abdominal contrast-enhanced computed tomography (CT) showed the target sign in the ascending colon. An enhanced nodule was detected at the lead point, suggesting the presence of a tumor (Fig. 1a, b). Colonoscopy showed a tumor at the lead point of the intestinal intussusception (Fig. 2a). Evidence of bowel wall edema and bowel obstruction was also seen (Fig. 2b). Biopsy revealed the tumor as a neoplastic lesion, suspected as NET. Immunohistochemical findings showed the tumor cells were positive for chromogranin A, synaptophysin, and CD56. The impression was NET G1.

**Fig. 3:**
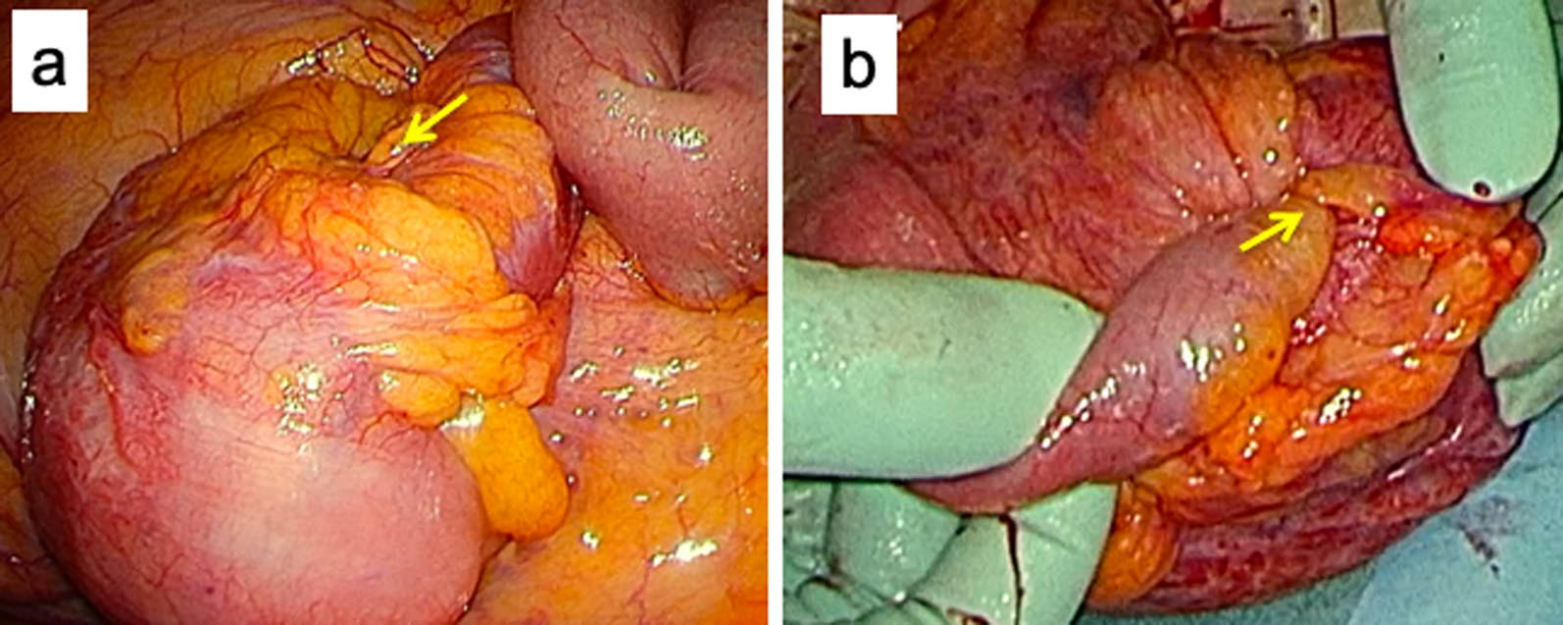
Surgical view shows evidence of intestinal intussusception (arrow). **a** Intracorporeal. **b** extracorporeal

**Fig. 4:**
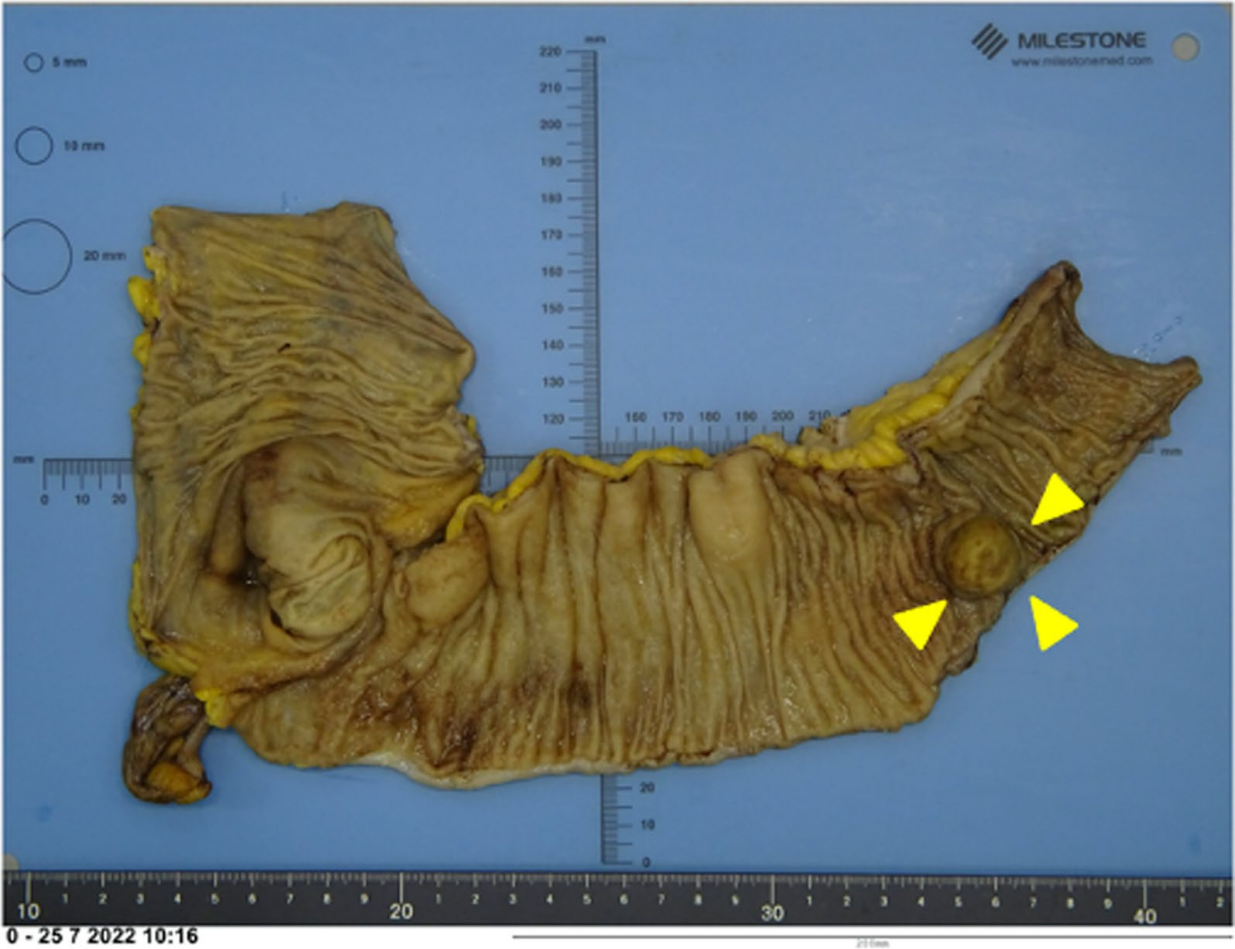
Resected specimen. A mass, approximately 2.1 cm in diameter, as a leading point for intestinal intussusception is detected 20 cm proximal to the ileocecal valve. Arrowhead: a lead point

**Fig. 5:**
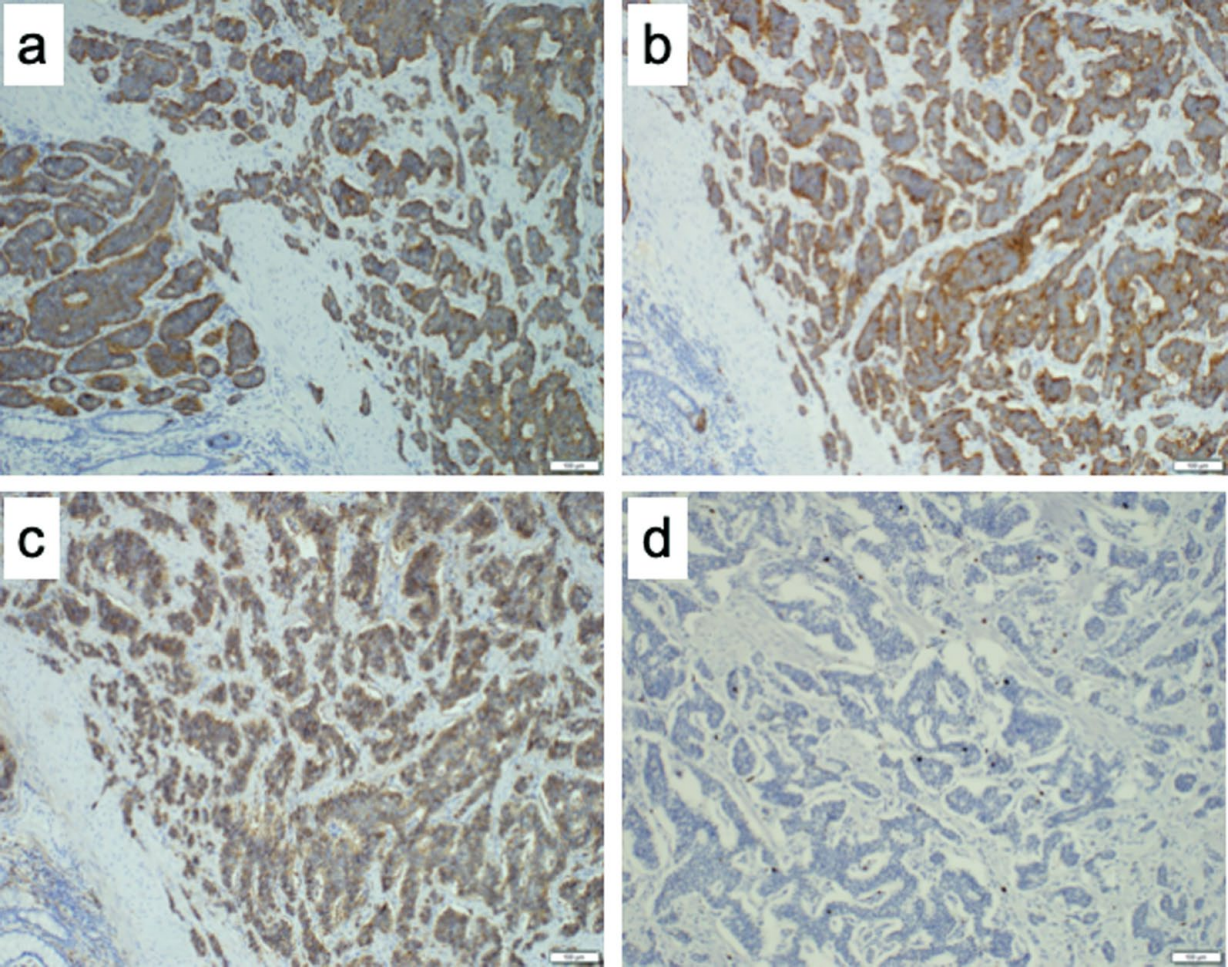
Immunohistochemistry findings of excised specimen. **a** Chromogranin A (×100), **b** synaptophysin (×100), **c** CD 56 (×100), **d** MIB-1 (×100)

After the induction of general anesthesia, an initial vertical incision was made at the umbilicus using the open technique. A Lap-Protector (Hakko Co., Nagano, Japan) was then inserted via the umbilical incision. EZ access (Hakko Co.) was used to insert two 5-mm trocars and a 12-mm trocar was placed at the Lap-Protector. Laparoscopic observation showed evidence of intestinal intussusception (Fig. 3a, b). The restoration of intestinal intussusception was not successful intraoperatively due to severe bowel wall edema. Single-incision laparoscopic ileocecal resection with D3 lymph node (LN) dissection, defined as removal of the main LNs at the root of the feeding vessels, was performed. The operation time was 138 min, and the blood loss was 5 ml. The patient was discharged 10 days postoperatively with no complications. Histological examination showed a tumor measuring 2.1 cm × 1.9 cm at the end of the ileum (Fig. 4). The tumor had invaded into the muscularis propria. Lymphatic and vascular invasion was present. The resection margins were negative. Immunohistochemical investigations revealed that tumor cells were positive for chromogranin A, synaptophysin, and CD56 (Fig. 5a–c). The impression was NET, well-differentiated G1, with 1 mitosis per 10 high-power fields, and MIB-1 0.5% (Fig. 5d). Lymph node involvement was seen in 2 of the 32 nodes investigated.

## Discussion

According to a PubMed search, this seems to represent the first case report to show the efficacy of the SILS for intestinal intussusception due to NET G1. This case showed two important clinical findings. First, the lead point of the intestinal intussusception was able to be detected preoperatively and NET G1 was diagnosed. Based on the tumor site, the appropriate range of LN dissection was then decided. Second, the single-incision laparoscopic approach with regional lymphadenectomy was able to be safely performed in a patient with intestinal intussusception due to NET G1, without any complications.

Intestinal intussusceptions can be categorized into four types according to the location: (i) enteric type, when the intestinal intussusception is limited to the small bowel; (ii) ileocolic type, when the ileum passes the ileocolic segment, but the appendix does not invaginate; (iii) ileocecal type, when the ileocecal portion invaginates into the ascending colon; and (iv) colocolonic (including colorectal) type, when the intestinal intussusception is limited to the colon and rectum, with no anal protrusion [8]. More than 90% of intestinal intussusceptions in adult are caused by a pathologic lead point, such as a polyp, benign or malignant tumor, Meckel’s diverticulum, colonic diverticulum, or postsurgical adhesion. These lead points are commonly discovered intraoperatively [9]. Reviewing the literature, 63–77.3% were tumor-related and 50–73.5% of these tumor-related lead points were malignant [8, 10]. The etiology of adult intestinal intussusception (ileocolic type) in the present case was tumor-related due to NET.

In adult intestinal intussusception, preoperative diagnosis of the etiology of intestinal intussusception is important to help guide treatment decisions. CT examination is the gold standard for diagnosing intestinal intussusception. Azar et al. reported that 78% of patients were accurately diagnosed intussusception by CT [1]. In our case, CT examination allowed preoperative identification of intestinal intussusception and colonoscopy examination revealed the lead point due to NET G1. Preoperative diagnosis of NETs is important for treatment as well as identifying intestinal intussusception. Surgical resection of an NET requires complete resection with regional lymphadenectomy, so optimal methods for lymphadenectomy are being investigated [11]. In the present case, we selected ileocecal resection with regional lymphadenectomy because of ileocolic-type intestinal intussusception due to NET G1. As above, preoperative assessment is an important aspect of surgical planning, helping surgeons identify patients requiring lymphadenectomy when the intestinal intussusception is associated with malignant tumor. We were able to preoperatively diagnose the NET G1 in this case.

We have described a case in which SILS was performed for adult ileocolic-type intestinal intussusception caused by NET G1. Conventionally, open surgery should be considered for treatment of adult intestinal intussusception, but several authors have recently reported that laparoscopic surgery for adult intestinal intussusception was useful and effective [12, 13]. Moreover, the short- and long-term safety of laparoscopic approach for colorectal cancer is well established. SILS represents a recent advance in MIS and has recently been shown to provide satisfactory oncological outcomes in patients with colon cancer [14–16]. In the present case, SILS with regional lymphadenectomy was successfully performed for intestinal intussusception caused by NET G1. In ileocecal-type intestinal intussusception, SILS have some advantages over the MIS: (1) reducing unnecessary number of ports, which may be also beneficial to wound care and cause less damage for patients, in cases with a narrow working space and significant ileus symptoms that may lead to conversion to laparotomy; (2) facilitating direct visualization and attempting to restoration of the intestinal intussusception from the umbilical incision because the lesion can be moved through mobilization to the working space in the abdominal cavity below the umbilicus; (3) facilitating direct visualization and attempting to restoration of the intestinal intussusception from the umbilical incision without mobilization, if an ileocecal region was not anchored to the retroperitoneum. The restoration of intestinal intussusception in this case was not successful intraoperatively, but the restoration of intestinal intussusception also leads to serve several functions, including reduction the extent of resection, setting an appropriate lymph node dissection area, reducing the surgical procedure, and allowing radical surgery for cancer. Thus, these points may be the advantage of SILS over MIS. As above, the experience gained in our case indicates that SILS may offer a useful therapeutic tool for selected cases of adult intestinal intussusception.

## Conclusion

SILS with regional lymphadenectomy can be safely performed for adult intestinal intussusception due to NET G1. This method may be useful for adult intestinal intussusception.

## Data Availability

Please contact author for data requests.

## Abbreviations

NETs: Neuroendocrine tumors
GEP-NETs: Gastroenteropancreatic neuroendocrine tumors
SILS: Single-incision laparoscopic surgery
MIS: Multiport laparoscopic surgery
CT: Computed tomography
LN: Lymph node
